# PepFect14 mediates the delivery of mRNA into human primary keratinocytes and *in vivo*

**DOI:** 10.3389/fphar.2023.1219761

**Published:** 2023-07-13

**Authors:** Kapilraj Periyasamy, Maria Maloverjan, Abhijit Biswas, Anu Remm, Martin Pook, Ana Rebane, Margus Pooga

**Affiliations:** ^1^ Institute of Biomedicine and Translational Medicine, University of Tartu, Tartu, Estonia; ^2^ Institute of Technology, University of Tartu, Tartu, Estonia

**Keywords:** cell-penetrating peptides, mRNA delivery, transfection, nanoparticles, skin inflammatory diseases, endocytosis inhibitors, internalization pathway

## Abstract

mRNA-based vaccines and candidate therapeutics have great potential in various medical fields. For the delivery of mRNA into target cells and tissues, lipid formulations are often employed. However, this approach could cause the activation of immune responses, making it unsuitable for the treatment of inflammatory conditions. Therefore, alternative delivery systems are highly demanded. In this study, we evaluated the transport efficiency and characteristics of cell-penetrating peptide PepFect14 (PF14) and mRNA nanoparticles in the presence of different additives. Our results show that all PF14-mRNA formulations entered cultured cells, while calcium chloride enhanced the transport and production of the encoded protein in HeLa and HaCaT cell lines, and polysorbate 80 did so in primary human keratinocytes. All formulations had similar physical properties and did not remarkably affect cell viability. By selectively blocking endocytosis pathways, we show that PF14-mRNA nanoparticles primarily entered HeLa cells via macropinocytosis and HaCaT cells via both macropinocytosis and clathrin-mediated endocytosis, while none of the blockers significantly affected the delivery into primary keratinocytes. Finally, subcutaneous injection of PF14-mRNA nanoparticles before inducing mouse irritant contact dermatitis resulted in the expression of a reporter protein without provoking harmful immune responses in the skin. Together, our findings suggest that PF14-mRNA nanoparticles have the potential for developing mRNA-based therapeutics for treating inflammatory skin conditions.

## 1 Introduction

The development of mRNA therapy has gained great attention as mRNA-based vaccines have become useful tools for controlling the spread and reducing the severity of the coronavirus disease 2019 (COVID-19) (Fang et al., 2022; Hussain et al., 2022). Accordingly, a large number of preclinical and clinical trials assessing mRNA-based candidate therapeutics have been initiated (Kulkarni et al., 2021; Sasso et al., 2022). Unlike DNA-based therapeutics, mRNA does not need to be transported into the cell nucleus, as the only step needed to get the desired product is translation, which makes mRNA an attractive tool for protein expression and reduces the risk of insertional DNA mutagenesis. Another advantage of mRNA is its ability to encode every human protein, so it can be potentially used for the treatment of a wide variety of conditions, with examples including cancer (Kübler et al., 2015), inherited metabolic disorders (Truong et al., 2019), Alzheimer’s disease (Lin et al., 2016), and heart injury (Sultana et al., 2017).

Lipid nanoparticles (LNPs) are widely used as a carrier system for cellular delivery of mRNA-based COVID vaccines and cancer vaccines currently in development (Bevers et al., 2022; Chen et al., 2022). LNPs are often slightly cytotoxic and immunogenic and therefore act as adjuvants, which is advantageous for vaccination (Alameh et al., 2021) and cancer immunotherapy (Zhang et al., 2021). However, there are many other applications where immune system activation is undesirable or harmful, for example, autoimmune conditions and chronic inflammatory diseases.

Cell-penetrating peptides (CPPs) are short peptides, consisting of less than 30 amino acid residues, that can effectively introduce a wide range of cargos into the cells, including different types of nucleic acids (Lehto et al., 2012; Veiman et al., 2013; Singh et al., 2018; Kurrikoff et al., 2021). CPPs are easily modifiable with specific amino acids to possess different activities (Baoum et al., 2012; Margus et al., 2012; Reissmann, 2014; Derakhshankhah and Jafari, 2018). For example, PepFect14 (PF14) is an amphipathic CPP that belongs to the PepFect family and is derived from transportan 10 (TP10) modified with stearic acid (Ezzat et al., 2011; Anko et al., 2012). As a result of the incorporation of non-proteinogenic amino acid ornithine, PF14 is less susceptible to serum proteases than its parent peptide (Ezzat et al., 2011), and stearyl moiety coupled to its N-terminus increases transfection efficiency (Futaki et al., 2001; Tönges et al., 2006; Mäe et al., 2009). PF14 is an efficient carrier with low cytotoxicity. It has been shown to effectively transduce various types of nucleic acids, including plasmid DNA (pDNA) (Veiman et al., 2013; Veiman et al., 2015), siRNA (Srimanee et al., 2018; Ervin et al., 2019), miRNA (Urgard et al., 2017; Carreras-Badosa et al., 2020), and antisense oligonucleotides (Ezzat et al., 2011; Cerrato et al., 2020; Luna Velez et al., 2022). Recently, in a study performed by Brand et al. (van den Brand et al., 2019), PF14 was utilized to introduce mRNA into tumor cells in a mouse model of ovarian cancer. Remarkably, the authors showed that the application of PF14-based nanoparticles led to high expression of mRNA-encoded protein in tumor-associated tissue, outperforming a lipid-based mRNA transfection reagent (van den Brand et al., 2019).

Although CPPs can successfully introduce nucleic acids to different cell types, there are still obstacles that can decrease the biological activity of the cargo. One of these is ineffective endosomal escape, which results in the destruction of the majority of cargo molecules in acidic endosomes (Turner et al., 2005; Gillmeister et al., 2011; Erazo-Oliveras et al., 2012). The endosomal escape can be improved by the incorporation of certain additives. For example, chloroquine significantly enhances the transport of nanoparticles by preventing endosomal acidification and, at higher doses, induces instability and rupture of the endosomal membrane (El-Andaloussi et al., 2007; El-Sayed et al., 2009; Lee et al., 2014). Calcium chloride (CaCl_2_) is another substance that has been shown to increase delivery efficiency and facilitate the endosomal escape of CPP-nucleic acid nanoparticles (Goldshtein et al., 2016). In addition, we have shown that magnesium chloride (MgCl_2_) has a similar effect to that of CaCl_2_, although it is usually less efficient (Maloverjan et al., 2022; Biswas et al., 2023). Another substance, a nonionic surfactant polysorbate 80 (PS80), has been shown to greatly enhance the transport of lipid-based nanoparticles into the brain (Li et al., 2018), including those carrying siRNA (Li et al., 2021) and pDNA (Attia et al., 2018). To the best of our knowledge, the effects of chloroquine, CaCl_2_, MgCl_2_, and PS80 on the PF14-mRNA nanoparticles have not yet been evaluated as well as there is very little known about the how these additives affect primary cell cultures.

Although it is recognized that endocytosis is the primary route for CPPs to enter the cells, the specific mechanism of internalization is not well known (Liu et al., 2013; Kondow-McConaghy et al., 2020; Ruseska and Zimmer, 2020; Trofimenko et al., 2021). Endocytosis can be divided mainly into macropinocytosis, clathrin-dependent, caveolin-dependent, or clathrin- and caveolin-independent endocytosis. Different types of endocytosis are utilized by cells to internalize nanoparticles with different chemical compositions. For instance, nanoparticles of PF14 and splice-correcting oligonucleotides are mainly internalized via macropinocytosis (Juks et al., 2015), while for PF14-pDNA nanoparticles, caveolin-dependent endocytosis is the primary route (Veiman et al., 2013). The delivery routes of PF14-mRNA have not been investigated previously.

In this study, we used PF14 to introduce mRNA-encoding reporter proteins into the immortal cell lines HeLa and HaCaT, primary human keratinocytes, and *in vivo* in a mouse model of irritant contact dermatitis (ICD). We evaluated the impact of chloroquine, CaCl_2_, MgCl_2_, and PS80 on the characteristics of PF14-mRNA nanoparticles and assessed the efficiency of transport and cell viability with these formulations. We also explored the cellular delivery routes of PF14-mRNA nanoparticles by blocking different endocytosis pathways generally utilized by CPP nanoparticles. Our findings demonstrate that PF14 can effectively deliver mRNA both *in vitro* and *in vivo*, providing the potential for designing therapeutic PF14-mRNA nanoparticles for the treatment of inflammatory conditions.

## 2 Materials and methods

### 2.1 Materials

mRNAs: Anti-reverse cap analogue (ARCA) mRNA encoding enhanced green fluorescent protein (EGFP), i.e., EGFP-mRNA (5-moUTP), ARCA Cy5-labeled EGFP-mRNA (5mo-UTP), and CleanCap mCherry-mRNA (5mo-UTP) were purchased from APExBIO Technology (Houston, TX, USA), and CleanCap Fluc-mRNA, i.e., luciferase-mRNA (5moU) from TriLink Biotechnologies (San Diego, CA, USA). The mRNA stock solutions (1 mg/mL) were maintained at −80°C.

PF14 (stearyl-AGYLLGKLLOOLAAAALOOLL-NH_2_) (PepScan, Lelystad, Netherlands and PepMic, Shanghai, China). 1 mM peptide stock solution was prepared in the mixture of ethanol, DMSO, and trimethylene carbonate (TMC) (90:9.6:0.4 v:v:v) and stored at −20°C. Luciferase substrate XenoLight D-Luciferin – K^+^ salt (LH2) was obtained from PerkinElmer (Waltham, MA, USA), CaCl_2_ and MgCl_2_ from Thermo Fisher Scientific (Waltham, MA, USA), PS80 from VWR (Radnor, PA, USA) and chloroquine from Applichem (Darmstadt, Germany). The cell lysis buffer contained 1% (v:v) Triton X-100, 50 mM Tris (pH 7.5), 150 mM NaCl, and 1 mM EDTA. Luciferase substrate solution contained 1 mM LH2, 25 mM DTT, 1 mM ATP, 25 μM coenzyme A, 5 mM MgSO_4_, 20 mM tricine, 1 mM EDTA, and 1 mM MgCO_3_.

### 2.2 Cell cultivation

HeLa (Expacy. Cellosaurus HeLa (CVCL_0030), n.d.) and human immortalized keratinocyte cell line HaCaT (Expacy. Cellosaurus HaCaT (CVCL_0038), n.d.) were cultured in Dulbecco’s Modified Eagle Medium (DMEM) (Corning, NY, USA) containing 4.5 g/L of glucose, 10% (v:v) of fetal bovine serum (FBS) and 1% (v:v) of penicillin-streptomycin solution (100 U/mL penicillin and 100 μg/mL streptomycin). Cells were grown on 6 cm cell culture dishes (Corning) at 37°C in a humid atmosphere that contained 5% of CO_2_ and were split every other day using trypsin-EDTA solution (Corning). For washing cells, Dulbecco’s Phosphate-Buffered Saline (DPBS) without calcium and magnesium was used. For experiments, cells were plated on 96-well plates (Thermo Fisher Scientific) at 1 × 10^4^ cells per well.

Pooled primary human epidermal keratinocytes (Promocell, Heidelberg, Germany) were cultured as previously described (Hermann et al., 2017). For the experiments, 4 × 10^4^ and 1 × 10^4^ cells were seeded in 24-well plates and 96-well plates, respectively, using a keratinocyte serum-free medium (KC-SFM) supplemented with recombinant epidermal growth factor (EGF), bovine pituitary extract (Gibco, Waltham, MA, USA), and antibiotic solution (5 U/mL penicillin and 5 μg/mL streptomycin).

### 2.3 PF14-mRNA nanoparticle formation and transfection

For the formation of nanoparticles, PF14 was mixed with mRNA at a charge ratio (CR) 2:1 in Milli-Q (MQ) water in 1/10th of the final volume if not stated otherwise. The final concentration of mRNA was 0.8 ng/μL and PF14 was 1 μM. After 15 min incubation at room temperature (RT), CaCl_2_, MgCl_2_, PS80, chloroquine, or their combinations were added if indicated, followed by 15 more min of incubation. After formation, nanoparticle-containing solutions (1/10th of the final volume) were added to the cell medium (9/10th of the final volume) and cells were incubated with the solutions at 37°C for 24 h, if not stated otherwise. All of the transfections were performed with cells plated either in 96- or 24-well plates with density of 50%–70%. A growth medium containing 1/10th (v:v) of MQ water was used as a negative control solution in all *in vitro* experiments.

### 2.4 Inhibition of endocytosis

Before the transfection, cells were pre-incubated for 30 min at 37°C with growth medium (9/10th of the final volume) that contained inhibitors of endocytosis at the following concentrations: 10 μM chlorpromazine, 50 μM nystatin, and 20 μM 5-(N-ethyl-N-isopropyl)-amiloride (EIPA). After pre-incubation, nanoparticle-containing solutions (1/10th of the final volume) were added and cells were incubated with inhibitors and nanoparticles for 4 h at 37°C. After this, solutions were replaced with a fresh growth medium, and the effects were measured after an additional 20 h of incubation.

### 2.5 Luminescence measurement

Cells were transfected with luciferase-mRNA for 24 h, then washed with PBS. To lyse the cells, 20 μL of cell lysis buffer was added and samples were incubated at −20°C until the solutions froze. Once thawed at RT, 70 μL of luciferase substrate solution was added per well, and the solutions were transferred to a white 96-well plate (Perkin Elmer, Waltham, MA, USA). The luminescence intensity was measured as previously described (Helmfors et al., 2015; Rocha et al., 2016) using the GloMAX 96 microplate luminometer (Promega, Madison, WI, USA).

### 2.6 Flow cytometry

The cells were transfected with nanoparticles of PF14 with mCherry-mRNA, EGFP-mRNA, or Cy5-mRNA for 24 h as in the protocol outlined in subsection 2.3. The cells were then detached by trypsinization, and the suspensions were diluted with a PBS buffer containing 0.5% BSA and 1 mM EDTA. Then, the plate was placed on ice. Next, cell nuclei were stained with 0.5 μg/mL of 6-diamidino-2-phenylindole (DAPI) in PBS buffer for 5 min to distinguish between live and dead cells. The flow cytometry analysis was conducted using an Attune NxT flow cytometer (Thermo Fisher Scientific) and 5000 events were analyzed for each well.

### 2.7 Cell viability assay

After 24 h of transfection with PF14-mRNA nanoparticles, cell viability was measured spectrophotometrically using the WST-8 assay kit (APExBIO Technologies) to determine the cytotoxicity of nanoparticles. Briefly, 10 µL of WST-8 solution per well was added after 24 h of transfection, and cells were incubated at 37°C for 3 h. Finally, the absorbance of the resultant solutions was measured at 450 nm, using an Infinite M200 PRO (Tecan, Männedorf, Switzerland) microplate reader. The percentage of viable cells was calculated using the following formula:

$$cell\;viability\;\left(\%\right)=\frac{absorbance\;of\;test\;compound-absorbance\;of\;blank}{absorbance\;of\;control-absorbance\;of\;blank}\;*100$$

### 2.8 Dynamic light scattering (DLS) analysis

Nanoparticles of mRNA and PF14 were prepared as described above and diluted 1:100 in MQ water (with a final volume of 1 mL). The hydrodynamic diameter and zeta potential of the nanoparticles were measured using the ZetaSizer Nano ZSP instrument (Malvern Panalytical, Malvern, UK).

### 2.9 Confocal microscopy

Cells were plated in a 24-well plate on cover glasses of 12 mm diameter (Menzel-Gläser, Braunschweig, Germany) 24 h prior to the transfection, which was carried out as described in subsection 2.3. After 24 h transfection, cells were washed with PBS and fixed by incubation with 4% paraformaldehyde (PFA) for 15 min at RT. Next, the cell nuclei were stained with 5 μg/mL of DAPI solution in PBS for 10 min at RT. Afterwards, the cells were washed with PBS and mounted onto glass slides with DAKO fluorescence mounting media (Dako Products, Agilent Technologies, CA, USA). For each condition, three specimens were prepared and at least three images per specimen were obtained. The images were taken using a confocal laser scanning microscope Olympus FV1200MPE (Olympus Corporation, Tokyo, Japan) with ×10 objective and ×60 oil objective at 1024 × 1024-pixel resolution. The images were captured at the wavelengths suitable for detection of DAPI (λex/em = 405/461 nm), EGFP (λex/em = 473/510 nm), and Cy5 (λex/em = 635/664 nm).

### 2.10 *In vivo* delivery of PF14-mRNA nanoparticles

Mice were maintained in the animal facility at the Institute of Biomedicine and Translational Medicine, University of Tartu, according to the institute’s regulations. Six-to-ten-week-old C57BL/6J mice were used in the experiments. All animal experiments were performed according to permission by the Animal Ethics Committee at the Estonian Ministry of Agriculture (license No. 117, 2018; No. 158, 2020). For subcutaneous injection, nanoparticles were formed initially by combining 25.6 µM PF14 and 42.5 ng/μL of EGFP-mRNA or EGFP encoding mRNA labeled with Cy5 in MQ water for 15 min at RT. Subsequently, if indicated, 3 mM CaCl_2_, or 0.05 mg/mL PS80 were added, followed by an additional 15 min incubation. Prior to injection, the resulting complexes were mixed with a 5% glucose solution. As a control, 20 µL of 5% glucose in PBS was used. To induce ICD, 20 μL of 0.2% phorbol 12-myristate 13-acetate (PMA) in acetone was applied topically. Ear thickness was measured with a Vernier digital caliper (Mitutoyo, Kawasaki, Japan) as previously described (Carreras-Badosa et al., 2020). For immunofluorescence analyses, mouse ears were fixed in 4% PFA for 24 h at 4°C. Afterwards, they were incubated in 30% sucrose for additional 24 h at 4°C before being embedded in Neg-50 cryo-medium tissue tek (Thermo Fisher Scientific). To perform confocal imaging, 10 μm thick sections of mouse ears were obtained from a cryotome. These sections were then permeabilized with 0.3% Triton X-100 in PBS for 10 min and incubated with 2% goat serum for 30 min at RT. The mouse ears were then stained with 10 μg/mL of EGFP polyclonal primary antibody (CAB4211, Thermo Fisher Scientific) followed by 10 μg/mL goat anti-rabbit IgG secondary antibody conjugated with Alexa Fluor 647 (A21245, Thermo Fisher Scientific). Specimens were also counterstained with DAPI and mounted with a fluorescent mounting medium (Dako Products). Finally, each slide was examined using an Olympus FV1200MPE confocal microscope (Olympus corporation).

### 2.11 RNA isolation, cDNA synthesis and RT-qPCR

Total RNA was purified using miRNeasy mini kit (Qiagen, Venlo, Netherland) following the manufacturer’s protocol. The RNA concentration and quality were evaluated with a NanoDrop 2000c spectrophotometer (Thermo Fisher Scientific). Subsequently, cDNA was synthesized from 400 to 600 ng of RNA using oligo-dT (TAG Copenhagen, Denmark), and RevertAid reverse transcriptase (Thermo Fisher Scientific). Quantitative PCR was performed on a Viia7 Real-time PCR system (Life Technologies, Carlsbad, California, USA) using 5× HOT FIREPol EvaGreen mix (Solis BioDyne, Tartu, Estonia). To normalize gene expression, HPRT was used, and ΔΔCt method was employed to calculate relative changes in gene expression.

### 2.12 Statistical analysis

Statistical analyses were performed using Prism 9.5.1 software (GraphPad Software, San Diego, CA, USA). Either one-way or two-way ANOVA test was used to compare mean values between studied groups or conditions, and the appropriate correction method was employed. In particular, Tukey’s multiple correction method was used when every mean was compared with every other mean, and Šidák multiple correction method was utilized for the comparison of selected sets of means, as indicated in the figure legends. The results were considered significant at *p* < 0.05.

## 3 Results

### 3.1 PF14 forms with mRNA and additives positively charged nanoparticles with suitable characteristics for cellular uptake

As the transport efficiency and safety of nanoparticles largely depend on the physicochemical characteristics, it is essential to optimize their size, charge, and chemistry (Desai, 2012; Sabourian et al., 2020). In addition, knowing the impact of particle size and charge on the uptake process, nanoparticles can be redesigned to accumulate maximally in the target areas with minimal side effects. Therefore, we first tested the effect of different additives on the size and charge of the PF14-mRNA nanoparticles using DLS analysis. For that, we formed nanoparticles of PF14 with four different mRNAs: mCherry-mRNA, luciferase-mRNA, EGFP-mRNA, and Cy5-labeled EGFP-mRNA (hereafter referred as Cy5-mRNA), using a charge ratio of 2:1 PF14:mRNA. As additives, PS80, chloroquine, CaCl_2_, and MgCl_2_ were added to the nanoparticles.

**FIGURE 1:**
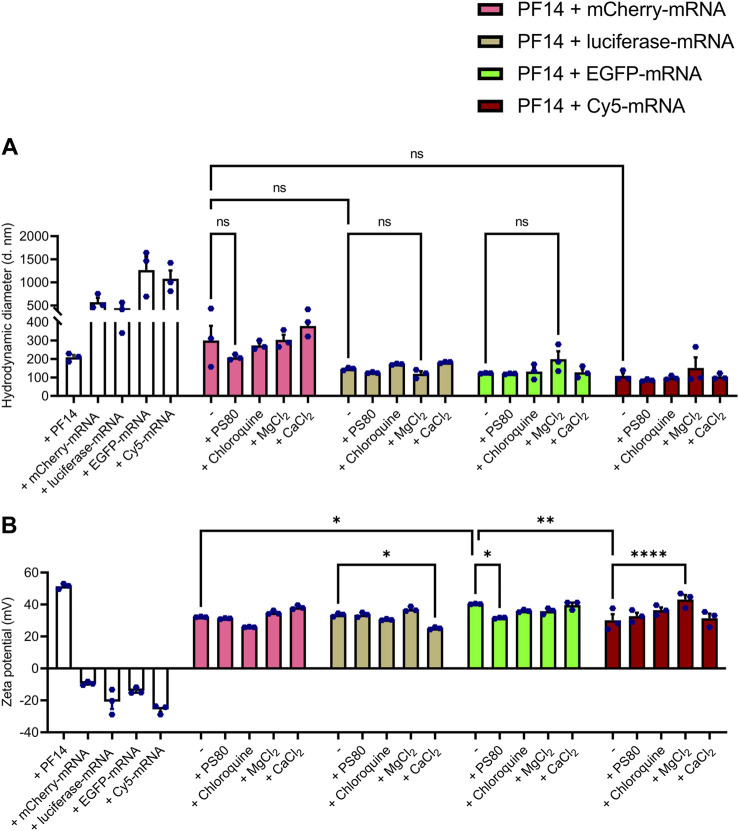
PF14-mRNA nanoparticles are with suitable characteristics for cellular delivery. (**A,B**) For DLS, nanoparticles were prepared using PF14 and four different mRNAs: mCherry, luciferase, EGFP and Cy5-mRNA at charge ratio (CR) 2:1. If indicated, nanoparticles were supplemented with PS80, chloroquine, MgCl_2_ and/or CaCl_2_. The white bars represent the treatments, where only PF14 or indicated mRNAs were added to the cells. Data are representative of three technical replicates and expressed as ± SEM, *n* = 3, one-way ANOVA with post-hoc Šidák test was used, ns – not significant, *p < 0.05, **p < 0.01, ****p < 0.0001.

DLS measurements revealed that the average hydrodynamic diameters of the particles formed by PF14 with mCherry, luciferase, EGFP and Cy5-mRNA were 300.1 nm, 146.6 nm, 123.1 nm, and 108.5 nm, respectively. This indicates that the different mRNAs, although having the same length, can have a slight influence on nanoparticle size (Figure 1A; Supplementary Table S1). Notably, PF14-EGFP-mRNA nanoparticles had a slightly higher zeta potential (40.3 mV) compared to those formed with mCherry (32.2 mV) and Cy5-mRNA (30.1 mV) as depicted in Figure 1B. Still, there were no significant changes in particle size when different additives were used for the formation of PF14-mRNA nanoparticles (Figure 1A). Furthermore, addition of CaCl_2_ to PF14-luciferase-mRNA (25.1 mV) and PS80 to PF14-EGFP-mRNA (31.6 mV) resulted in reduced zeta potential, while the addition of MgCl_2_ to Cy5-mRNA nanoparticles led to a mild increase in zeta potential (43.2 mV) (Figure 1B; Supplementary Table S1). Our results also showed that when PF14 was absent, all tested mRNAs exhibited a negative charge, with values ranging from −9.4 mV to −25.6 mV. However, when PF14 was added, all resulting nanoparticles had a positive charge, with values ranging from 25.1 mV to 43.2 mV, as shown in Figure 1B and summarized in Supplementary Table S1. Overall, these results demonstrate that all of the formed nanoparticles possessed suitable characteristics for further experiments.

### 3.2 The addition of CaCl_2_ enhances the transport efficiency of PF14-mRNA nanoparticles into cell lines without affecting cell viability

**FIGURE 2:**
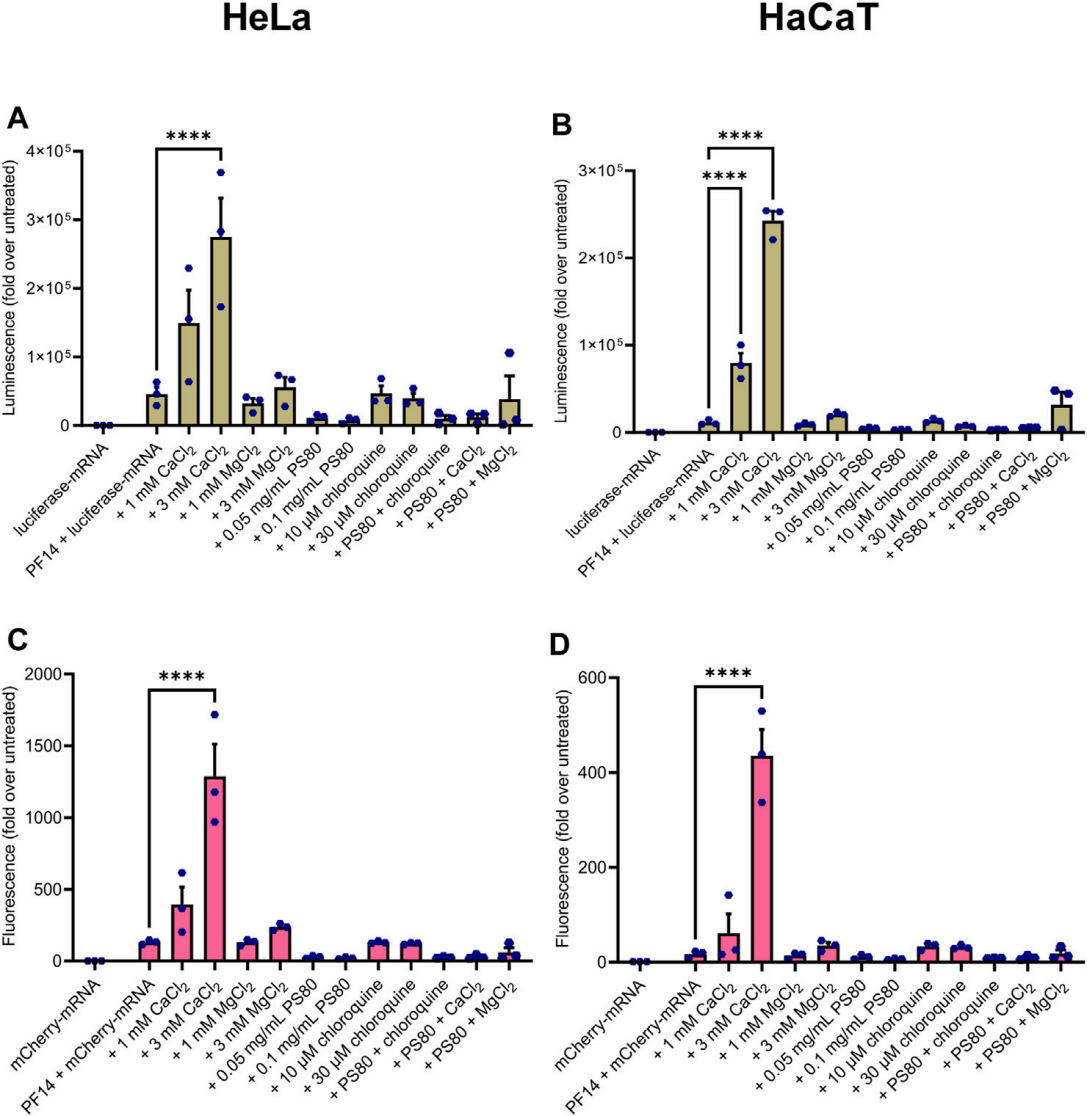
CaCl_2_ increases the productive delivery of PF14-mRNA nanoparticles in human cell lines. Nanoparticles were formed from PF14 and luciferase-mRNA **(A,B)** or mCherry-mRNA **(C,D)** and after 15 min, the additives were added. HeLa **(A, C)** or HaCaT **(B, D)** cells were incubated with the nanoparticles for 24 h, and then the reporter protein expression was measured. Data are representative of three independent experiments, with each data point representing the mean of three replicates within a single experiment. Data were analyzed with one-way ANOVA with post-hoc Šidák test, ****p < 0.0001.

Next, we assessed whether the PF14-mRNA nanoparticles formed with luciferase-mRNA and mCherry-mRNA in the presence and absence of additives induced the expression of reporter proteins in a cancer-derived cell line HeLa and immortalized keratinocyte cell line HaCaT. In addition, we investigated the impact of nanoparticles on the viability of these two cell lines. All of the nanoparticles induced protein expression to a certain extent, as seen in Figures 2A–D. However, among the tested compounds, only CaCl_2_ significantly enhanced the expression of both luciferase (Figures 2A, B) and mCherry (Figures 2C, D) in both tested cell lines. Notably, the addition of PS80, chloroquine, and MgCl_2_ to the nanoparticles did not affect the expression of the reporter protein in either cell line. While none of the nanoparticles were significantly toxic to HeLa cells (Supplementary Figure S1A), PS80 considerably affected the viability of HaCaT cells (Supplementary Figure S1B). In summary, PF14 effectively delivers mRNA into cell lines, and addition of CaCl_2_ into the nanoparticles enhances their effect in HeLa and HaCaT cells without affecting cell viability.

### 3.3 PF14 mediates transfection of mRNA into primary human keratinocytes

**FIGURE 3:**
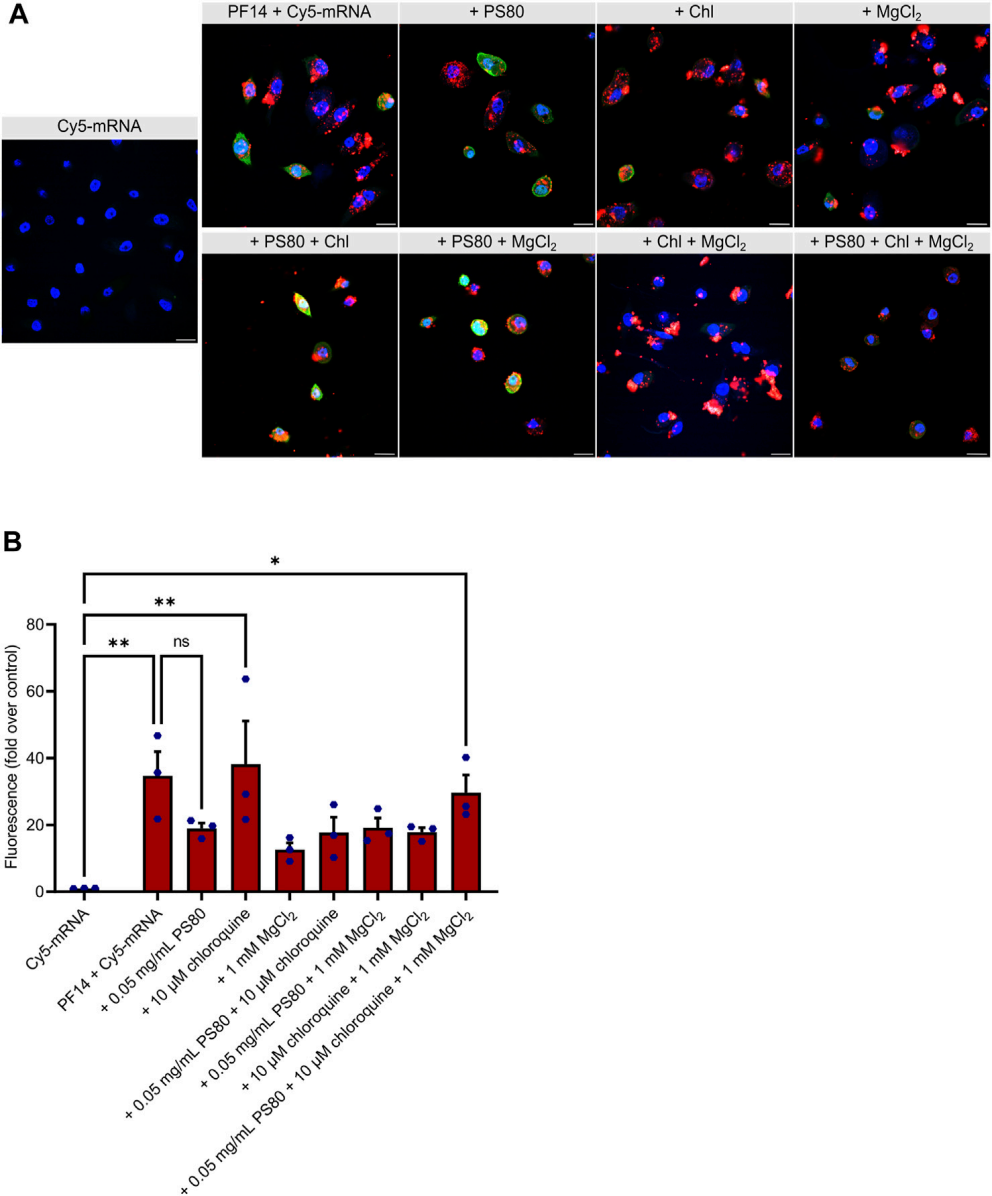
PF14 delivers mRNA into primary human keratinocytes. **(A,B)** Keratinocytes were transfected for 24 h with PF14-Cy5-mRNA (red) nanoparticles (CR 2:1) expressing EGFP (green) with or without additives: PS80, MgCl_2_, chloroquine, and their combinations. **(A)** Cell nuclei were stained with DAPI (blue) and images were obtained at ×60 magnification with oil immersion. Chl – chloroquine, scale bar = 20 µm. **(B)** For quantification, confocal images were acquired at ×10 magnification and the mean fluorescence intensity of the Cy5 signal (red) was measured using ImageJ software and normalized with corresponding DAPI intensity. Data are representative of three independent samples for each condition (*n* = 3). Data are expressed as mean ± SEM, one-way ANOVA with Šidák multiple correction test was used, ns – not significant, *p < 0.05, **p < 0.01.

We next examined the transport efficiency of PF14-Cy5-mRNA nanoparticles into primary human keratinocytes using confocal microscopy. As depicted in Figure 3A and Supplementary Figure S2, after 24 h, all nanoparticles were capable of transporting Cy5-mRNA into the cytoplasm of keratinocytes. Due to its reported ability to induce keratinocyte differentiation (Xie et al., 2005; Mahanty et al., 2019), CaCl_2_ was excluded from experiments with keratinocytes. Quantitation analysis of images revealed that none of the additives significantly enhanced or reduced the delivery (Figure 3B). However, we observed a less efficient delivery when PS80 or MgCl_2_ were added, as measured by the Cy5 signal alone. Furthermore, the EGFP signal was relatively weak in most of the conditions, indicating inefficient translational of Cy5-mRNA in the keratinocytes upon application of the PF14-Cy5-mRNA nanoparticles (Figure 3A). In conclusion, our results demonstrate that all tested combinations of additives exhibited similar levels of PF14-Cy5-mRNA transportation into primary human keratinocytes.

**FIGURE 4:**
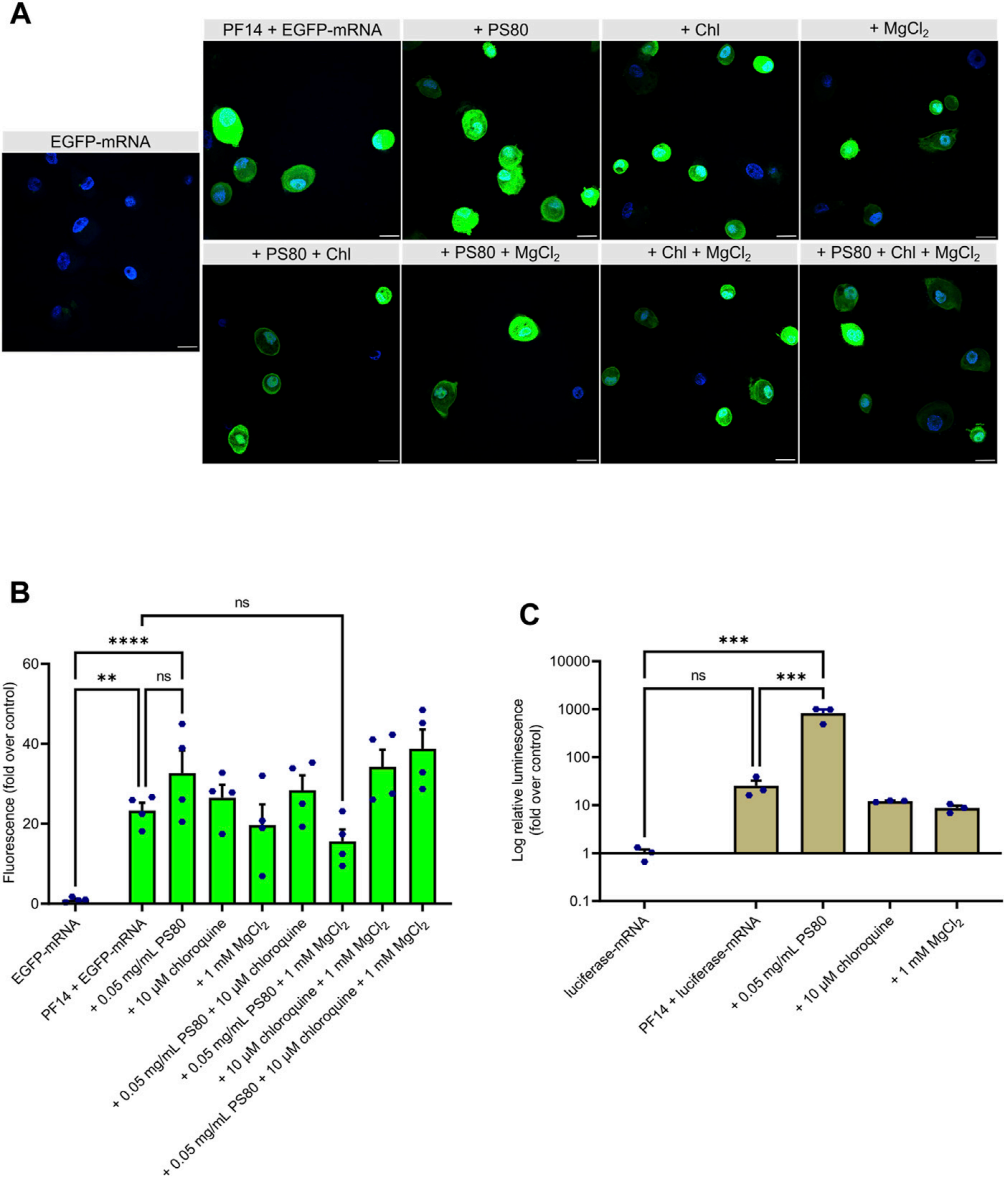
PS80 enhances expression of mRNA-encoded reporter protein in primary human keratinocytes. **(A,B)** Keratinocytes were incubated for 24 h with PF14-EGFP-mRNA nanoparticles prepared with and without additives. **(A)** Cell nuclei were stained with DAPI and images were obtained at ×60 magnification with oil immersion. Chl – chloroquine, scale bar = 20 µm. **(B)** For quantification, confocal images were acquired at ×10 magnification. The mean fluorescence intensity of EGFP (green) was measured using ImageJ software and normalized to corresponding DAPI (blue) intensity. Data are representative of four independent samples in each condition (*n* = 4). **(C)** Keratinocytes were incubated with the nanoparticles prepared of luciferase-mRNA, PF14, and the indicated additives. After 24 h, cells were harvested for luminescence intensity measurement. Data are representative of three independent samples per condition (*n* = 3). **(B–C)** Data are expressed as mean ± SEM, one-way ANOVA with Šidák multiple comparison test was used, ns – not significant, **p < 0.01, ***p < 0.001, ****p < 0.0001.

Next, we used EGFP-mRNA for formation of similar nanoparticles. Our results demonstrate that transfection of all PF14-based EGFP-mRNA nanoparticles led to the EGFP expression in keratinocytes (Figures 4A, B). Interestingly, among additives, PS80 significantly increased the expression of the reporter protein in keratinocytes (Figure 4B; Supplementary Figure S3), which was further confirmed with the use of luciferase-mRNA (Figure 4C). In addition, our flow cytometry data also demonstrated that PS80 greatly increased the EGFP expression after 24 h of transfection, as shown in Supplementary Figure S3B. Importantly, the addition of PS80 did not alter the fluorescence intensity of Cy5, indicating a specific effect on reporter protein expression after the delivery (Supplementary Figure S3B). However, the inclusion of MgCl_2_ and chloroquine did not appear to enhance the efficiency of PF14-EGFP-mRNA nanoparticles (Figures 4B, C). It should be noted that the nanoparticles containing PS80 had a substantial impact on cell viability compared to the PF14-mRNA nanoparticles alone in both keratinocytes (Supplementary Figure S1C) and in HaCaT cells (Supplementary Figure S1B). In addition, we observed that naked Cy5-mRNA and PF14-mRNA nanoparticles reduced the viability of keratinocytes depending of the additives included, while HaCaT cells were less affected, and HeLa cells showed no influence. These results collectively demonstrate that primary human keratinocytes can be successfully transfected with PF14-mRNA nanoparticles. However, PF14-mRNA nanoparticles have a greater impact on the viability of primary human keratinocytes compared to cell lines.

### 3.4 PF14-mRNA nanoparticles are internalized by cells via macropinocytosis and clathrin-mediated endocytosis

Despite the uptake of CPPs has been reported for a wide range of cell types, the specific mechanism of internalization is not well understood and varies considerably depending on cell type, cargo, and the physicochemical features of nanoparticles (Ruseska and Zimmer, 2020). To investigate the internalization pathway of PF14-mRNA nanoparticles with or without additives, CaCl_2_ or PS80 were chosen since they were effective in cell lines and keratinocytes, respectively. Then, HeLa, HaCaT, and keratinocytes were treated with endocytosis inhibitors, such as chlorpromazine, which inhibits clathrin-mediated endocytosis, EIPA, which inhibit macropinocytosis, and nystatin, which inhibits caveolin-mediated endocytosis (Ivanov, 2008; Rennick et al., 2021).

**FIGURE 5:**
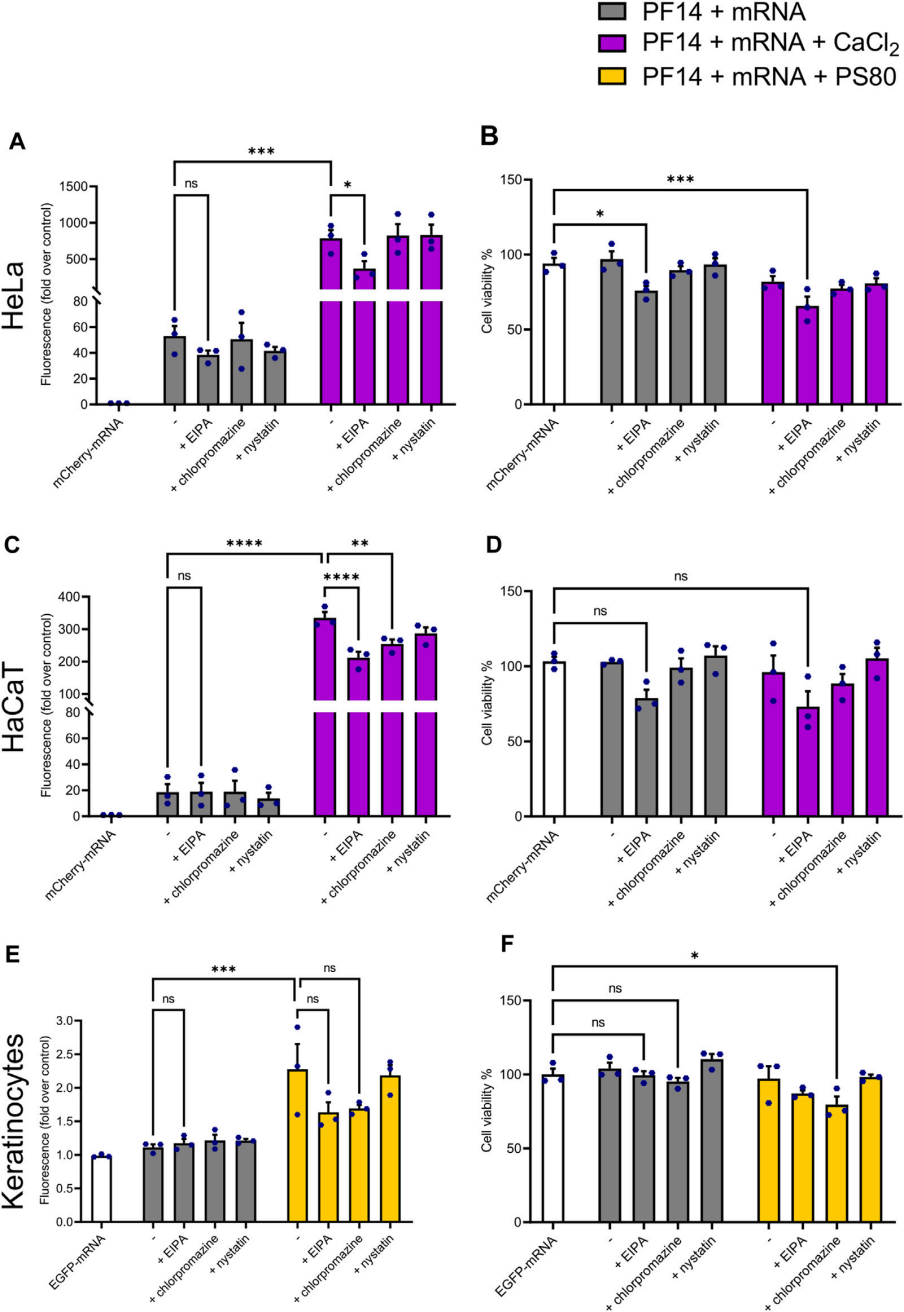
PF14-mRNA nanoparticles are mainly transported via macropinocytosis or clathrin-mediated endocytosis into HeLa and HaCaT cell lines. **(A, C, E)** The influence of reagents blocking different routes of endocytosis on PF14-mRNA transfection in HeLa and HaCaT cells and primary human keratinocytes. **(A)** HeLa and **(C)** HaCaT cells were transfected with mCherry-mRNA and the effects were measured using flow cytometry. **(E)** The mean fluorescence intensity was measured in keratinocytes transfected with EGFP-mRNA using flow cytometry. **(B, D, F)** The effect of inhibitors on the viability of **(B)** HeLa cells, **(D)** HaCaT cells, and **(F)** primary human keratinocytes. **(A–F)** Data are representative of three independent samples (*n* = 3). Data expressed as mean ± SEM, one-way ANOVA with Šidák multiple comparison test was used. Asterisks indicate statistically significant differences between two indicated datasets, ns – not significant, *p < 0.05, **p < 0.01, ***p < 0.001, ****p < 0.0001.

Our data demonstrate that EIPA significantly reduced the expression of a reporter protein in HeLa cells, confirming that PF14-mCherry-mRNA nanoparticles predominantly translocate into HeLa cells via macropinocytosis (Figure 5A). In the case of HaCaT cells, PF14-mCherry-mRNA nanoparticles containing CaCl_2_ were internalized via macropinocytosis and clathrin-mediated endocytosis, as application of EIPA and chlorpromazine limited the expression of a reporter protein significantly (Figure 5C). In contrast, none of the blockers significantly altered the transport of the PF14-EGFP-mRNA nanoparticles in the absence or presence of PS80 in primary keratinocytes, although EIPA and chlorpromazine apparently caused a slight reduction of EGFP expression (Figure 5E). It should be noted, that even though the media was changed after 4 h of endocytosis inhibitor treatment, the viability of cells was reduced under certain conditions. Specifically, EIPA treatment decreased the viability of HeLa cells (Figure 5B), while also exhibiting a tendency to affect the viability of HaCaT cells (Figure 5D). Additionally, chlorpromazine had an impact on the viability of keratinocytes as shown in Figure 5F.

### 3.5 PF14 transports mRNA *in vivo* in a mouse model of ICD upon local administration

**FIGURE 6:**
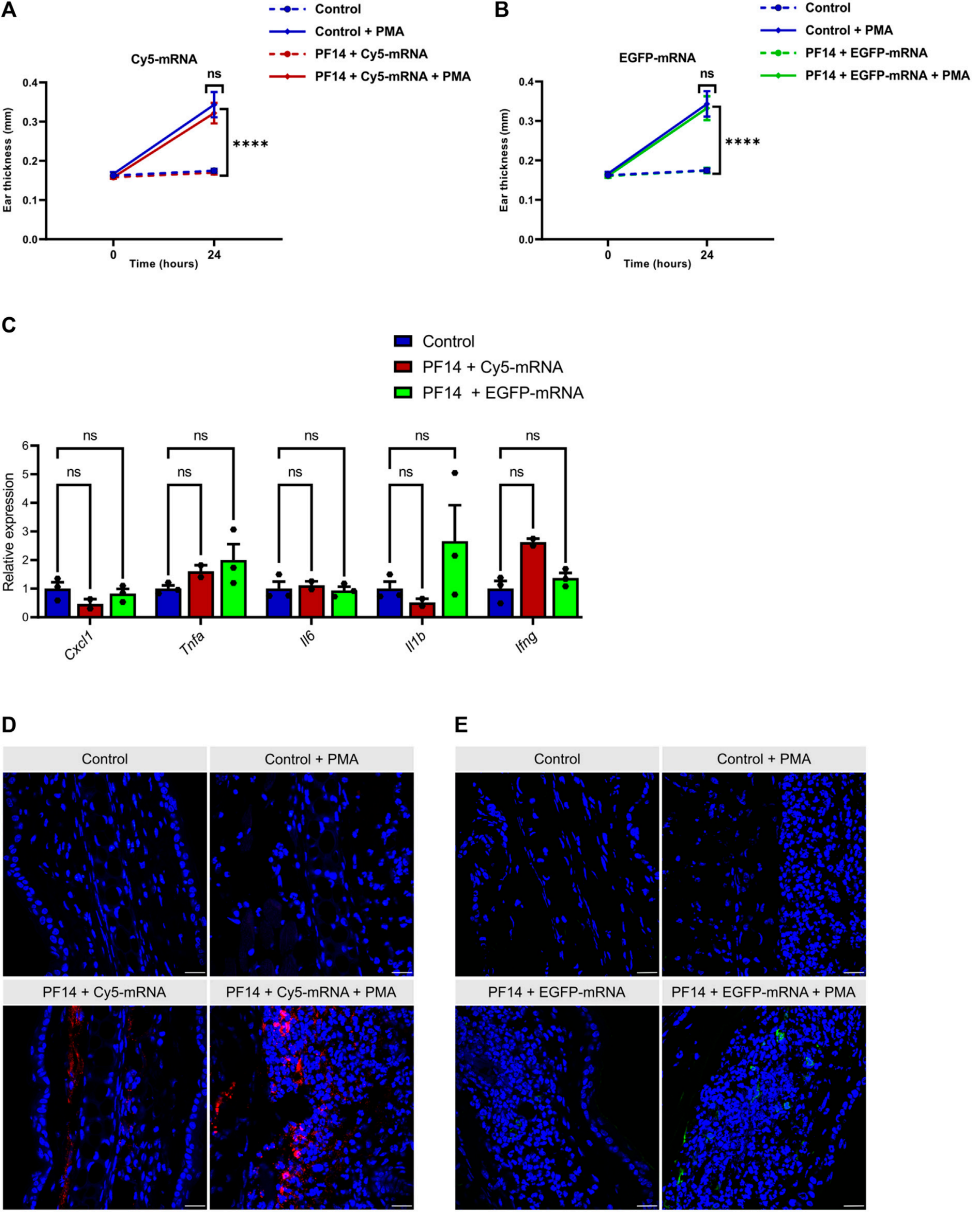
PF14 induces cellular transport of mRNA *in vivo*. For each injection, nanoparticles consisting of 850 ng of Cy5-mRNA or EGFP-mRNA at a CR of 2:1 PF14:mRNA in 20 µL of 5% glucose solution were used. **(A,B)** Ear thickness was measured at three sites of each ear at time points 0 h and 24 h (*n* = 4). **(C)** Relative expression of pro-inflammatory cytokines, including *Cxcl1*, *Tnfa*, *Il6*, *Il-1b*, and *Ifng*, was measured by RT-qPCR. The data were normalized to housekeeping gene HPRT (*n* = 3). **(A–C)** Data are expressed as mean ± SEM, two-way ANOVA with Tukey’s multiple comparison test was used, ns – not significant, ****p < 0.0001. **(D–E)** The confocal microscopy images show the distribution of mRNA and the expression of the reporter protein in PMA-treated and control ears. The tissue was counterstained with DAPI (blue). **(D)** The red signal represents the fluorescence of Cy5-mRNA. **(E)** Ear sections were stained with EGFP polyclonal antibody, followed by goat anti-rabbit Alexa Fluor 647 IgG staining. The red color was manually changed to green pseudo-color and it represents the expression of EGFP. Scale bar = 100 μm.

Next, we evaluated the efficacy of PF14 in transporting mRNA into mouse ears through subcutaneous injection. To mimic the changes in delivery conditions that occur during inflammation, where tight junctions between cells open, we induced ICD in a subgroup of mice. This was done by applying 0.2% PMA topically to the mouse ears, as previously described (Carreras-Badosa et al., 2020). We employed Cy5-mRNA to study the distribution of nanoparticles, and EGFP-mRNA to evaluate cellular uptake of administered nanoparticles and subsequent protein expression. The injection of nanoparticles was carried out 1 hour before the induction of ICD. As expected, the thickness of mouse ears treated with PMA increased significantly, indicating ongoing tissue inflammation (Figures 6A, B). It is important to note that application of PF14-mRNA nanoparticles (with both Cy5-mRNA and EGFP-mRNA) did not result in any ear swelling, as shown in Figures 6A, B. Additionally, there were no significant changes in cytokines related to inflammation such as *Cxcl1*, *Tnfa*, *Il6*, *Il1b*, and *Ifng*, as shown in Figure 6C, indicating that there was no provocation of inflammation. However, there was a slight tendency towards an increase in *Il1b* expression in the PF14-EGFP-mRNA injected group and an increase in *Ifng* in the PF14-Cy5-mRNA injected group (Figure 6C). Upon subcutaneous injection of the nanoparticles, Cy5-mRNA was visible in both untreated ears and PMA-treated ears, with apparently more spread distribution of Cy5 in the latter case (Figure 6D). Furthermore, we also detected EGFP fluorescence signal near the injection site and found more EGFP-positive cells in the PMA-treated ears compared to the control (Figure 6E). However, neither CaCl_2_ nor PS80 improved the delivery of mRNA or the expression of a reporter protein *in vivo* (Supplementary Figure S4).

## 4 Discussion

For the delivery of mRNA-based vaccines and candidate therapeutics for cancers, lipid formulations are often employed. However, this approach causes in most cases the activation of immune responses and is therefore not suitable in large number of conditions, where immune system responses should be suppressed. Therefore, alternative methods for mRNA transport into target tissues and cells are still in great demand. Accordingly, in the current study, we aimed to generate PF14-based nanoparticles that are safe and effective in cell culture and in the mouse model of ICD. To optimize the nanoparticle composition, several compounds were tested as additives, including CaCl_2_, MgCl_2_, chloroquine, and PS80.

First, we analyzed the physical properties of PF14-mRNA nanoparticles formed with or without additives using DLS. When taken alone, mRNA forms in water structures/aggregates with highly polydisperse nature and a hydrodynamic diameter over 500 nm. However, when complexed with PF14, it is condensed into nanoparticles with a diameter of 100–300 nm. We observed that the additives did not significantly affect the hydrodynamic diameter of PF14-mRNA nanoparticles and that all formulations had a positive zeta potential. Even though PS80 lowered zeta potential of used nanoparticles to some extent, it did not reduce the uptake or expression from delivered mRNA in keratinocytes. It would be also interesting to characterize PF14-mRNA nanoparticles with TEM, however, as the used additives interfered with the standard negative staining of nanoparticles specimens (data not shown), further studies are needed to work out alternative staining methods to visualize PF14-mRNA nanoparticles with supplements.

Next, we performed cell culture experiments to evaluate the transfection efficiency of differently prepared nanoparticles. We showed that all formulations were able to transport mRNA into the cells, as evidenced by the expression of the reporter proteins encoded by mRNA. Interestingly, in HaCaT and HeLa cells, the addition of CaCl_2_ significantly improved the productive delivery of mRNA, while PS80, chloroquine, and MgCl_2_ had no significant effect. Previous studies have demonstrated that CaCl_2_ and MgCl_2_ enhance the biological effect of SCO, siRNA, and pDNA (Lam and Cullis, 2000; Baoum et al., 2012; Maloverjan et al., 2022; Biswas et al., 2023). In addition, it has been shown that CaCl_2_ improves nanoparticle delivery by increasing endosomal escape (Maloverjan et al., 2022), facilitating the formation of nanoparticles with serum components (Hori et al., 2015), and promotes crosslinking between peptides and nucleic acids (Baoum et al., 2009). We have previously observed that MgCl_2_ is beneficial in the transportation of ASOs in cell lines (Maloverjan et al., 2022; Biswas et al., 2023), however, the effect of Mg^2+^ seems to be weaker than that of Ca^2+^ in the case of siRNA delivery. For example, Ca^2+^ ions increased the delivery of siRNA nanoparticles, whereas Mg^2+^ had no effect, although they formed nanoparticles with similar physical features (Goldshtein et al., 2016). Chloroquine, which did not have positive effect on PF14-mRNA nanoparticles, is known to promote endosomal escape and enhance siRNA knockdown (Du Rietz et al., 2020) and oligonucleotide-mediated splicing correction (El-Andaloussi et al., 2006). These results collectively indicate that the effect of different additives also depends on nucleic acid used in the formation of nanoparticles, as the length, different modifications and secondary structure of siRNA/mRNA influence nanoparticles characteristics and endosomal escape.

In experiments with primary human keratinocytes, we used MgCl_2_, chloroquine and PS80 as the additives. Although showing the most promising results in cell lines, CaCl_2_ had to be excluded from experiments with keratinocytes, as it is known to induce cell differentiation (Xie et al., 2005; Mahanty et al., 2019). Similar to immortal cell lines, all used PF14-mRNA nanoparticle formulations were able to deliver mRNA into keratinocytes. However, the transfection of PF14-mRNA nanoparticles into primary keratinocytes had a greater effect on cell viability. It is possible that primary keratinocytes are not only more sensitive to nanoparticles but also to the mRNA used, which suggests that therapeutic mRNAs need to be carefully designed to avoid an immune response. It should also be noted that while PS80 improved the efficiency of mRNA transfection, it also had a negative impact on cell viability. Previous studies demonstrated that PS80 increased cellular internalization and improved efficiency of plasmid DNA in immortal cell lines, potentially due to its ability to enhance cell membrane fluidity and activate endocytosis (Tahara et al., 2010). We speculate that PS80 may exert similar effects in the case of keratinocytes, but this can also recognize as danger signal. Furthermore, it is important to note that while all the additives were included in the nanoparticles before incubation with the cells, the varying effects of these additives in cell lines compared to keratinocytes could also be due the influence of different cell culture media during the transfection process.

Several previous studies have focused on improving CPP based delivery systems for nucleic acids. For example, Abdelhamid et al., in 2020 achieved enhanced transfection of plasmid, SCO, and siRNA by successfully incorporating zeolitic imidazolate framework-8 (ZIF-8), a hybrid porous material into nucleic acid and PF14 nanoparticles (Abdelhamid et al., 2020). Additionally, Bell et al., in 2018 demonstrated that transfection efficiency of the CPP-mRNA nanoparticles was enhanced by using toll-like receptor antagonist E6446 (Bell et al., 2018). Furthermore, it was shown that the cellular delivery of hydrophilic CPPs could be improved when pyrenebutyrate was used as an additive (Guterstam et al., 2009). More recently, it was found that coating CPP-nucleic acid complexes with chondroitin conjugated with mannitol increased the penetration with low cytotoxicity. (Kumari et al., 2022). Further investigation is needed to comprehensively understand the effects of these additives in mRNA nanoparticles.

Although it is known that CPPs enter cells via endocytosis, the exact internalization mechanism is not fully understood and may vary depending on CPP properties, type of cargo, cell type, etc. (Ruseska and Zimmer, 2020). To investigate which endocytosis pathway is used by the cells for internalization of PF14-mRNA nanoparticles, we used endocytosis pathway inhibitors, such as chlorpromazine, which inhibits clathrin-mediated endocytosis, nystatin for inhibiting caveolin-mediated endocytosis, and EIPA for inhibiting macropinocytosis. Although the two cell lines used in the study, HeLa and HaCaT, showed some differences, macropinocytosis appeared to be the main mechanism by which PF14-mRNA nanoparticles entered both cell types. However, none of the blockers distinguishably changed the transport of the PF14-mRNA-PS80 nanoparticles into primary keratinocytes, although EIPA and chlorpromazine had a modest effect, which was not statistically significant. This can be explained by the limitations of the used method as multiple factors may affect the results in primary cells more than in cell lines, including cell culture medium composition, cell seeding density, and passage number (Sasso et al., 2018; Francia et al., 2019).

Finally, we transfected mRNA *in vivo* using local subcutaneous injection into the ears of mice. As in the case of inflammation delivery conditions change, we used the mouse model of ICD to generate an inflammatory environment. The particular model was chosen as a short “proof-of-concept” model that we had previously used successfully to test anti-inflammatory effect of miRNA nanoparticles formed with PF6 or NictFect71 (Urgard et al., 2016; Carreras-Badosa et al., 2020). Currently, inflammatory skin diseases are treated either with various topical treatments, systemic steroids, or biologicals. However, as these methods either demand continuous application, may cause adverse effects, or are not always efficient (Tausend et al., 2014; Weidinger and Novak, 2016; Renert-Yuval and Guttman-Yassky, 2018), there is still a need for novel modalities. We propose that local administration of anti-inflammatory synthetic mRNA at the site of interest would be a possible alternative option, which has several advantages, as the patient’s own recipient cells translate the mRNA into a functional protein, for example, to a certain cytokine or receptor inhibitor, that then acts as a therapeutic. Our results with Cy5-mRNA indicate that in the case of subcutaneous injection, PF14-mRNA nanoparticles are visible and spread in the vicinity of injection site during at least 24 h. Moreover, our preliminary results indicate that injections of PF14-mRNA nanoparticles do not cause any unwanted immune response at the site of injection as ear thickness and the expression of pro-inflammatory cytokines did not significantly change compared to the control injection. Though, as we detected reporter protein in a relatively low number of cells, there is a need for further optimization of the nanoparticle formulation to achieve more efficient delivery, endosomal escape, and the resulting translation of mRNA. Another limitation of our study is that we did not use mRNA that encodes a therapeutically relevant protein. Further studies are needed to test how effective can be nanoparticles containing mRNA that encodes either anti-inflammatory cytokines or receptor antagonists for the suppression of inflammatory responses.

## 5 Conclusion

In the current study, we demonstrate that PF14 is capable of delivering mRNA into cultured cells and when injected subcutaneously, *in vivo* in a mouse model of ICD. Among the tested additives, the addition of CaCl_2_ to the PF14-mRNA nanoparticles enhanced the expression of the mRNA-encoded reporter protein in HaCaT and HeLa cells and PS80 was beneficial in keratinocytes. Importantly, most of the PF14-mRNA nanoparticle formulations did not have significant impact on the viability of the studied cell types and did not cause ear swelling in mice. Our study is novel as it shows, for the first time that PF14-mRNA nanoparticles can be delivered into human primary keratinocytes and inflamed skin. In addition, we assessed first time the effect of chloroquine, CaCl_2_, MgCl_2_, and PS80 on mRNA transfection and delivery pathways. Our findings suggest that the use of PF14 for delivery of potentially therapeutic mRNA holds promise for the treatment of inflammatory conditions in the skin, such as chronic inflammatory skin diseases. Further studies are needed to test whether PF14-mRNA nanoparticles that contain mRNA encoding anti-inflammatory proteins are effective, as well as improvement of the nanoparticle formulations is needed.

## Data Availability

The original contributions presented in the study are included in the article/Supplementary Material, further inquiries can be directed to the corresponding authors.
