# Supplementary material for: PepFect14 mediates the delivery of mRNA into human primary keratinocytes and in vivo
Source: Front Pharmacol. 2023 Jul 13;14:1219761. doi: 10.3389/fphar.2023.1219761 (PMC10374019; doi:10.3389/fphar.2023.1219761)
Supplement: Supplementary file 1 [file Table2.DOCX]

Supplementary Material

PepFect14 mediates the delivery of mRNA into human primary keratinocytes and *in vivo*

Kapilraj Periyasamy^1†^, Maria Maloverjan^2†^, Abhijit Biswas^2^, Anu Remm^1^, Martin Pook^1^, Ana Rebane^1*^, and Margus Pooga^2*^

^1^Institute of Biomedicine and Translational Medicine, University of Tartu, Tartu, Estonia

^2^Institute of Technology, University of Tartu, Tartu, Estonia

*** Correspondence:**Margus Pooga
[margus.pooga@ut.ee](mailto:margus.pooga@ut.ee)

Ana Rebane
[ana.rebane@ut.ee](mailto:ana.rebane@ut.ee)

† These authors contributed equally to this work and share the first authorship

| Sample | Size (by intensity) | | Zeta potential |
| --- | --- | --- | --- |
|  | **Avg. d.nm** | **Avg. PDI** | **mV** |
| PF14 | 209.2 ± 22.07 | 0.45 ± 0.04 | 51.4 ± 1.7 |
| mCherry-mRNA | 572.5 ± 157.3 | 0.64 ± 0.19 | -9.4 ± 1.2 |
| luciferase-mRNA | 440.3 ± 113.6 | 0.44 ± 0.07 | -20.8 ± 7.8 |
| EGFP-mRNA | 1265.9 ± 504.3 | 0.8 ± 0.23 | -14.1 ± 2.0 |
| Cy5-mRNA | 1077.6 ± 314.2 | 0.74 ± 0.16 | -25.6 ± 2.9 |
| PF14 + mCherry-mRNA | 300.1 ± 137.5 | 0.52 ± 0.12 | 32.2 ± 0.29 |
| PF14 + mCherry-mRNA + PS80 | 208.8 ± 14.9 | 0.47 ± 0.04 | 31.2 ± 0.4 |
| PF14 + mCherry-mRNA + chloroquine | 273.3 ± 24.2 | 0.37 ± 0.02 | 25.8 ± 0.2 |
| PF14 + mCherry-mRNA + MgCl_2_ | 303.8 ± 7.5 | 0.55 ± 0.11 | 34.9 ± 1.22 |
| PF14 + mCherry-mRNA + CaCl_2_ | 378.8 ± 50.0 | 0.53 ± 0.13 | 38.2 ± 1.3 |
| PF14 + luciferase-mRNA | 146.6 ± 7.9 | 0.46 ± 0.02 | 33.5 ± 1.1 |
| PF14 + luciferase-mRNA + PS80 | 125.7 ± 4.1 | 0.43 ± 0.02 | 33.5 ± 1.5 |
| PF14 + luciferase-mRNA + chloroquine | 171.8 ± 4.1 | 0.49 ± 0.04 | 30.5 ± 0.46 |
| PF14 + luciferase-mRNA + MgCl_2_ | 119.9 ± 23.3 | 0.50 ± 0.01 | 37.0 ± 1.6 |
| PF14 + luciferase-mRNA + CaCl_2_ | 181.5 ± 4.8 | 0.46 ± 0.01 | 25.1 ± 0.8 |
| PF14 + EGFP-mRNA | 123.1 ± 2.0 | 0.37 ± 0.08 | 40.3 ± 0.3 |
| PF14 + EGFP-mRNA + PS80 | 119.9 ± 3.2 | 0.44 ± 0.01 | 31.6 ± 0.5 |
| PF14 + EGFP-mRNA + chloroquine | 131.4 ± 41.1 | 0.45 ± 0.13 | 36.0 ± 0.8 |
| PF14 + EGFP-mRNA + MgCl_2_ | 199.4 ± 73.7 | 0.35 ± 0.03 | 35.9 ± 1.9 |
| PF14 + EGFP-mRNA + CaCl_2_ | 127.8 ± 31.0 | 0.33 ± 0.05 | 39.7 ± 2.7 |
| PF14 + Cy5-mRNA | 108.5 ± 26.4 | 0.40 ± 0.13 | 30.1 ± 6.8 |
| PF14 + Cy5-mRNA + PS80 | 85.1 ± 5.7 | 0.57 ± 0.11 | 32.7 ± 3.7 |
| PF14 + Cy5-mRNA + chloroquine | 98.1 ± 11.3 | 0.35 ± 0.08 | 36.4 ± 3.1 |
| PF14 + Cy5-mRNA + MgCl_2_ | 152.0 ± 98.5 | 0.47 ± 0.07 | 43.2 ± 4.8 |
| PF14 + Cy5-mRNA + CaCl_2_ | 104.7 ± 15.7 | 0.32 ± 0.08 | 31.4 ± 5.1 |

**Supplementary Table S1.** Hydrodynamic diameter and zeta potential of particles prepared from PF14 and mRNA with different additives as measured by dynamic light scattering (DLS).

**
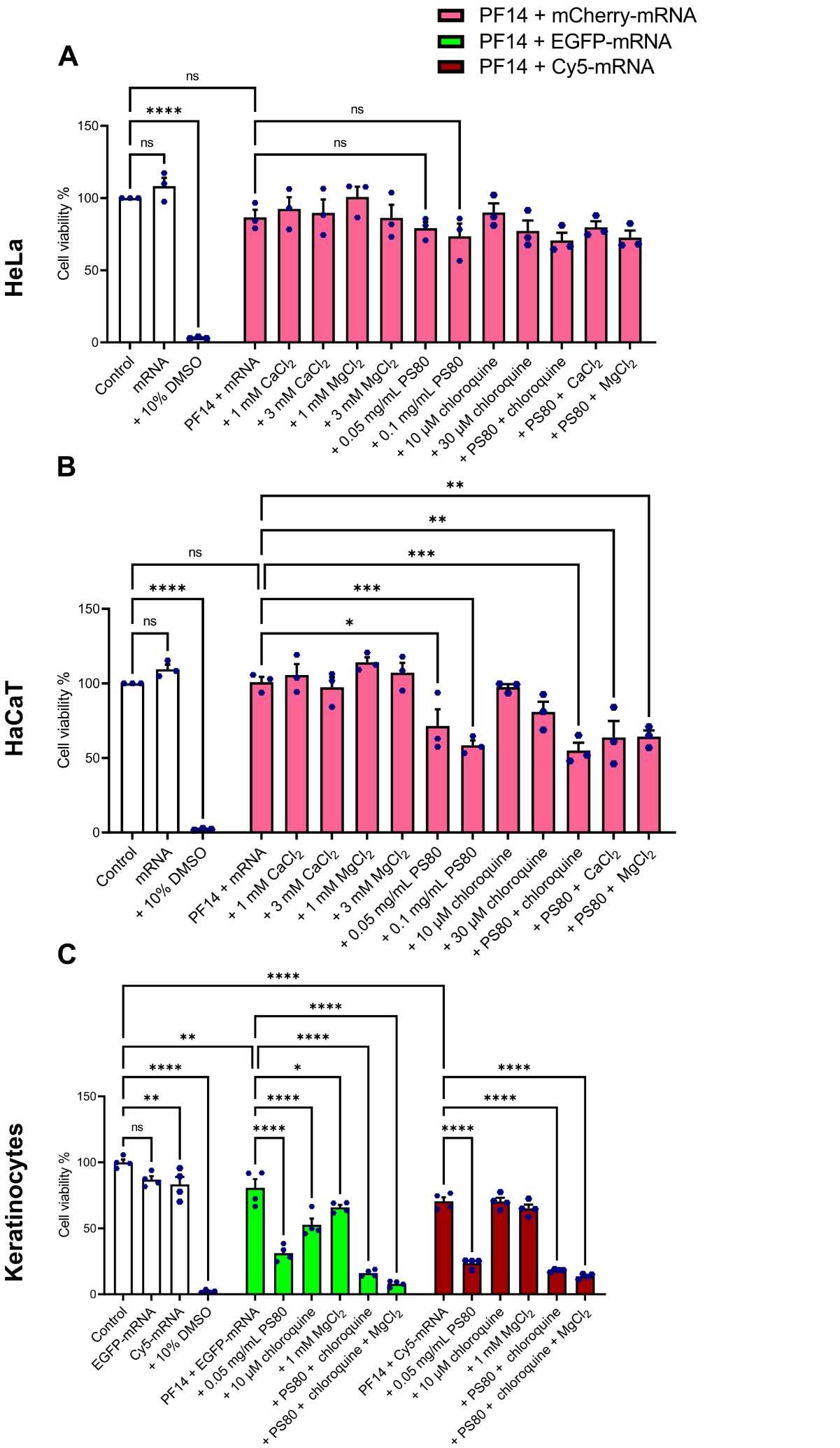
**

**Supplementary Figure S1.** The influence of nanoparticles on cell viability in HeLa (**A**) and HaCaT (**B**) cell lines and in primary human keratinocytes (**C**). Data are expressed as mean ± SEM, one-way ANOVA with Šidák multiple comparison test was used, ns – not significant, *p < 0.05, **p < 0.01, ***p < 0.001, ****p < 0.0001.


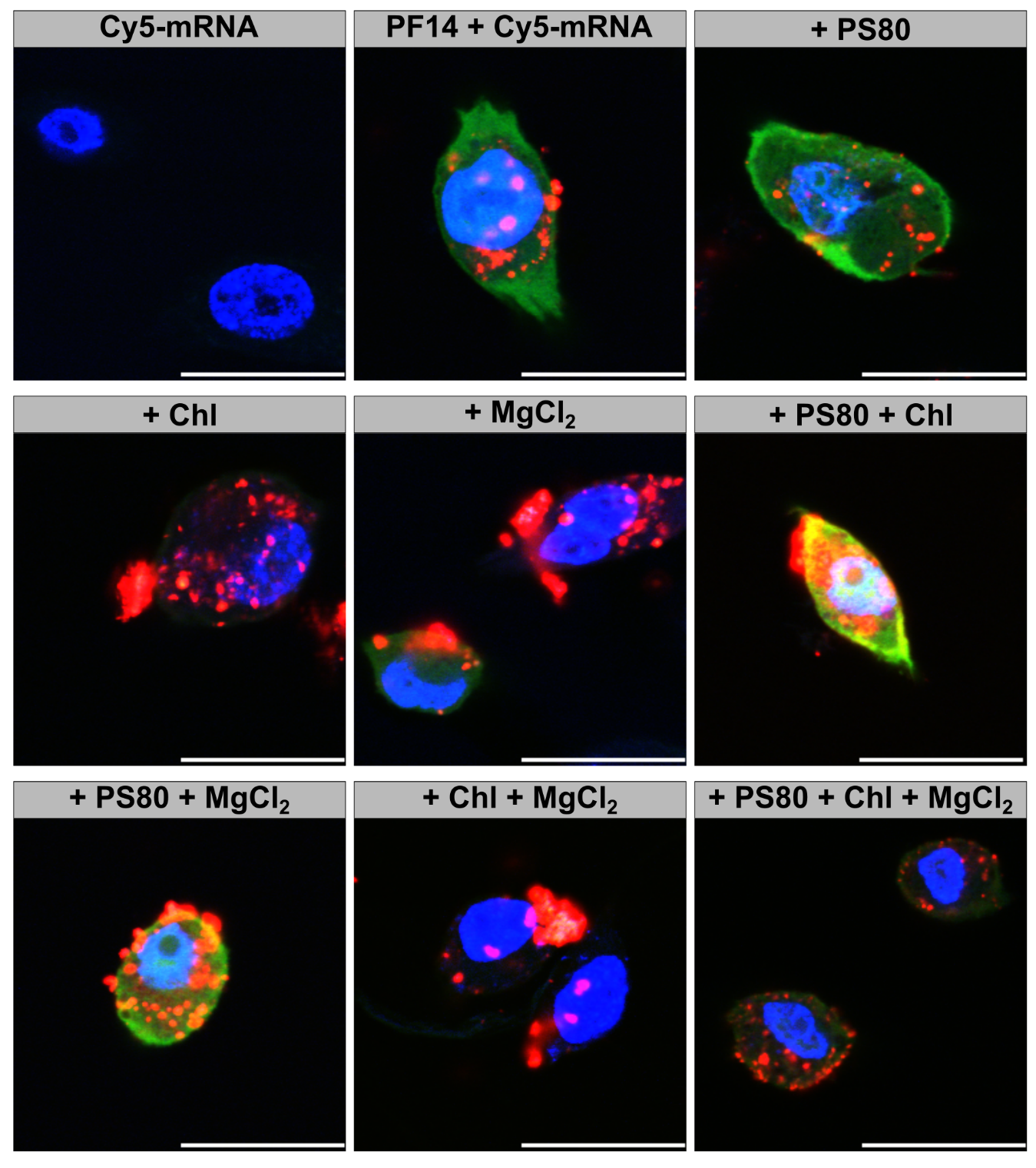


**Supplementary Figure S2.** PF14 transports mRNA into primary human keratinocytes. Keratinocytes were transfected with PF14-Cy5-mRNA nanoparticles (CR 2:1) expressing EGFP (green) with or without additives: PS80, MgCl_2_, chloroquine, and their combinations. Cell nuclei were stained with DAPI (blue) and images were obtained at 60× magnification with oil immersion. Chl – chloroquine, scale bar = 20 µm.


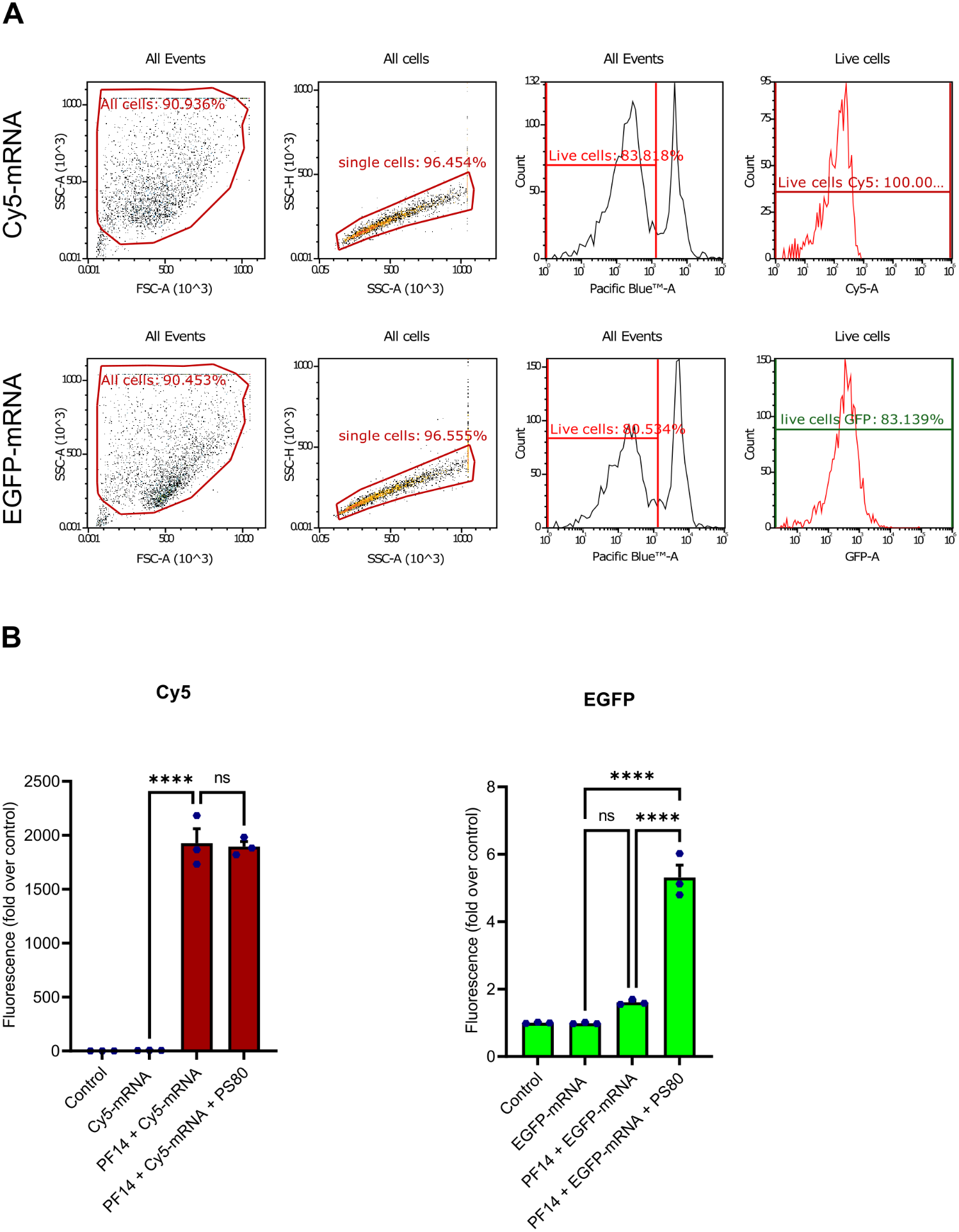


**Supplementary Figure S3.** The addition of PS80 to nanoparticle leads to an increased expression of the reporter protein in keratinocytes. Keratinocytes were transfected with PF14 and Cy5-mRNA and/or EGFP-mRNA nanoparticles followed by flow cytometry. (**A**) Representative graphs showing flow cytometry gating strategies. (**B**) Data are represented as mean ± SEM of three independent samples in each group (n=3), one-way ANOVA with Šidák multiple comparison test was used, ns – not significant, ****p < 0.0001.


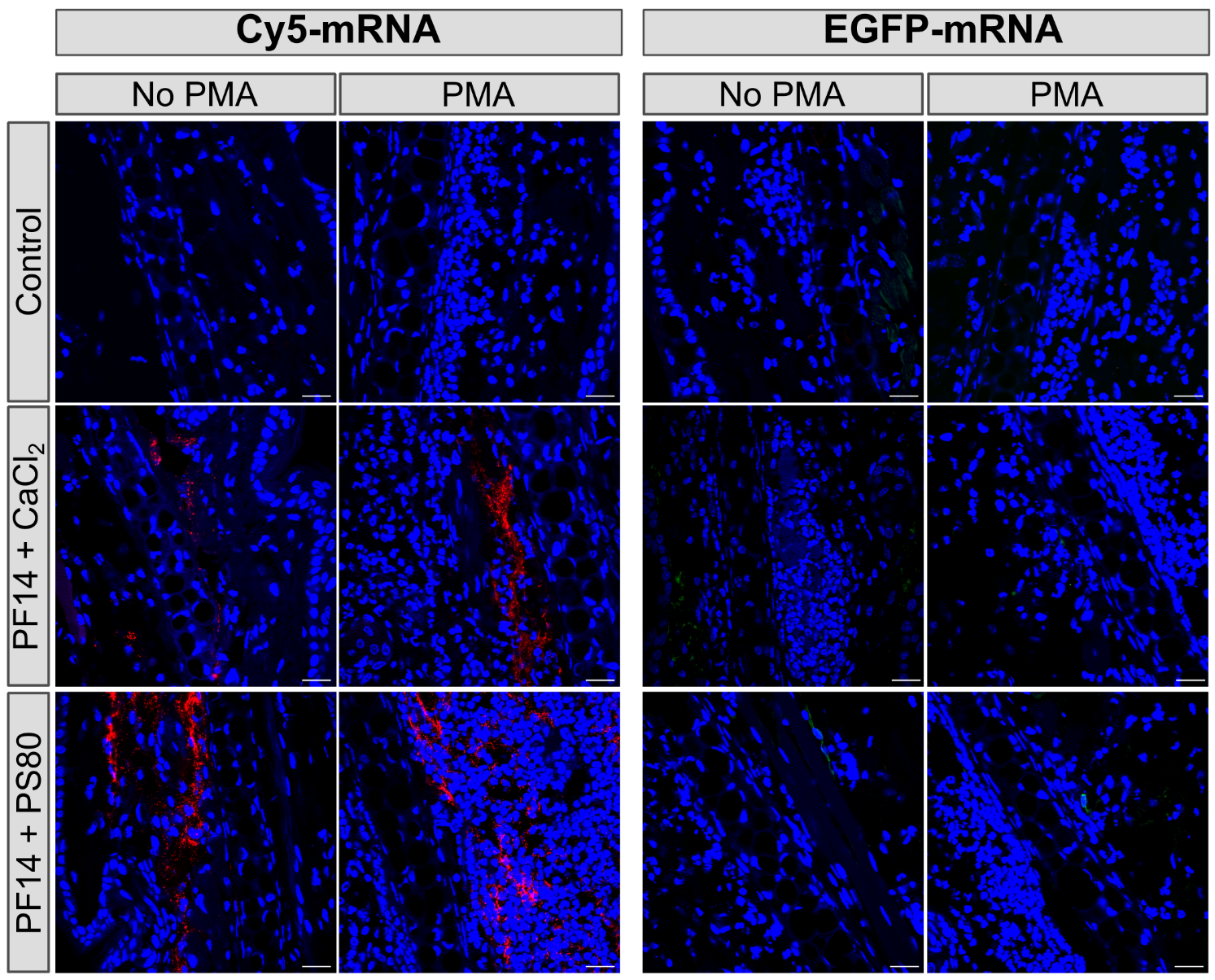


**Supplementary Figure S4.** The addition of PS80 or CaCl_2_ to nanoparticles did not improve productive delivery *in vivo*. 850 ng of Cy5-mRNA or EGFP-mRNA nanoparticles at a CR of 2:1 PF14:mRNA in the presence of indicated additives in 20 µL of 5% glucose solution were injected into mouse ears. The confocal microscopy images show the distribution of mRNA and the expression of the reporter protein in PMA-treated and control ears. The tissue was counterstained with DAPI (blue). In the left panel, the red signal represents the fluorescence of Cy5-mRNA. In the right panel, ear sections were stained with EGFP polyclonal antibody, followed by goat anti-rabbit Alexa Fluor 647 IgG staining. The red color was manually changed to green pseudo-color. Scale bar = 100 μm.
